# The ATHENA Study: Evaluating the impact of an educational intervention on Greek midwives’ knowledge and attitudes toward LGBTQ+ reproductive and perinatal care

**DOI:** 10.18332/ejm/211383

**Published:** 2025-10-31

**Authors:** Angeliki Antonakou, Eleni Theodoridou, Kalliopi Gkougkousidou, Vicentia C. Harizopoulou

**Affiliations:** 1 Department of Midwifery Science, School of Health Sciences, International Hellenic University, Thessaloniki, Greece; 2 Department of Midwifery, Faculty of Health and Caring Sciences, University of West Attica, Athens, Greece

**Keywords:** attitudes, knowledge, midwifery education, sexual and gender minorities, LGBTQ+

## Abstract

**INTRODUCTION:**

Inclusive reproductive and perinatal midwifery care for LGBTQ+ individuals requires adequate knowledge and positive attitudes. This study evaluated the impact of a structured educational intervention on Greek midwives' short- and long-term knowledge, and attitudes toward LGBTQ+ individuals and related health issues.

**METHODS:**

A longitudinal pre- and post-intervention study was conducted within the ATHENA Study in Greece. Seventy midwives attended a 3-hour, small-group session combining lecture, case-based discussion, and group dialogue on inclusive and gender-affirming care. Knowledge and attitude scores (reflecting attitudes toward LGBTQ+ individuals and patients) were assessed at baseline, post-intervention, and follow-up at 6 months, using the Greek PKSGMH survey. Analyses used Friedman’s test with Bonferroni correction (p<0.05).

**RESULTS:**

Data from 70 midwives (mean age 42.1 years, 97.1% women) were analyzed. Most were heterosexual (87.1%), married (62.9%), and had LGBTQ+ acquaintances (75.7%); only 18.6% had prior training. Post-intervention attitude scores toward gay men and lesbians decreased significantly (p=0.016 and p=0.011), indicating more positive attitudes, with improvements sustained at 6 months (p>0.999). Attitudes toward transgender people improved (p=0.034), remaining stable (p>0.999). Total and patient specific attitude scores decreased post-intervention (p=0.003 and p<0.001), sustained at follow-up (p>0.999). Knowledge scores increased (p=0.048 and p<0.001) and remained higher at 6 months (p=0.011 and p<0.001). Affirmative practice scores improved post-intervention (p=0.043) but not long-term (p=0.189).

**CONCLUSIONS:**

A brief educational intervention significantly improved midwives’ knowledge and attitudes toward LGBTQ+ individuals, with most effects sustained for six months. Embedding LGBTQ+-inclusive education in midwifery programs is essential for equitable reproductive and perinatal care.

## INTRODUCTION

Access to inclusive, gender-affirming and equitable reproductive and perinatal midwifery care remains a critical challenge for LGBTQ+ populations worldwide^1^. Despite growing recognition of health disparities and the increasing demand for reproductive equality, LGBTQ+ individuals often encounter barriers such as provider bias, pervasive discrimination, lack of cultural competency, and inadequate training in sexual and gender minority health^2,3^. These barriers are mounting mainly due to pervasive cis-heteronormative ideologies and practices within maternal and newborn care systems, which traditionally perceive pregnancy as an exclusively cisgender female experience^1^. These obstacles can negatively impact patient experiences, clinical outcomes, and access to and trust in healthcare systems during preconception, pregnancy, childbirth and postpartum care^2,4,5^. Midwives, as frontline providers in reproductive and perinatal care, are particularly well-positioned to address these disparities and to function as key advocates for inclusive and equitable care of LGBTQ+ populations^1,6^. Comprehensive, tailored education and training for midwives is consistently emphasized as crucial interventions to promote a deeper understanding of the unique needs, health risks and challenges faced by LGBTQ+ individuals in reproductive and perinatal care and to support diverse family structures^1-3,5,7^.

Moreover, while there is substantial published evidence that LGBTQ+ cultural-competency trainings consistently improve healthcare providers’ knowledge, attitudes, skills, and affirming practices, longitudinal evaluations remain scarce, limiting inferences about sustainability over time^8-13^. Existing literature suggests that midwives generally have good intentions and are motivated to learn strategies to better care for LGBTQ+ individuals^1,6,7,14^. However, gaps in knowledge and limited midwives’ exposure to LGBTQ+ health issues, contribute to heteronormative assumptions and suboptimal individual-midwife interactions, highlighting the urgent need for structured educational interventions, targeting attitudes, knowledge, and clinical skills in both curricula and continuing professional development programs^1-4,7,10,14-18^.

Despite the growing international body of evidence, research on midwives’ preparedness to provide care to LGBTQ+ populations in Greece is limited. Only one study has examined both knowledge and attitudes within the Greek context^14^, and systematic interventions to address these gaps are missing. Moreover, to our knowledge, no published work has evaluated the long-term sustainability of training effects, further underscoring the need for well-designed educational programs that both enhance competencies and assess their retention over time to promote inclusive reproductive and perinatal care.

The present study, which was carried out as part of the ATHENA Study, aims to assess the impact of a structured educational intervention on the short-term (immediately after the intervention) and long-term (six months later) knowledge and attitudes of Greek midwives toward LGBTQ+ individuals and LGBTQ+ health issues.

## METHODS

The present longitudinal interventional study employed a pre- and post-intervention design. It was carried out in Greece, from October 2024 to May 2025, as part of the ATHENA Study^19^, a research initiative supported by the Hellenic Foundation for Research and Innovation (H.F.R.I.) under the ‘Basic Research Financing’ call, which is part of the National Recovery and Resilience Plan ‘Greece 2.0’, funded by the European Union NextGenerationEU. The study protocol was approved by the International Hellenic University’s Research Ethics Committee (protocol number 31/2024- 09/04/2024).

### Recruitment

All registered midwives working in Greece, who were willing to participate, were considered eligible to participate voluntarily in the study. Participants were also free to withdraw at any time without consequences.

An open call for participation was published on the official website of the ATHENA Study^19^, in conjunction with the announcement of three scheduled educational interventions. In addition to the website announcement, formal invitations were sent to regional midwifery associations nationwide, with a request to disseminate the information to their members to maximize outreach.

The educational interventions were organized in three cities (Athens, Thessaloniki, and Ptolemaida), selected based on both population size (the capital and the second-largest city) and the presence of University Departments of Midwifery. The educational intervention was delivered on three separate days during November 2024, one in each city, and 70 midwives in total attended.

The announcement invited midwives not only to attend the educational interventions but also to complete online questionnaires on LGBTQ+ individuals and patients, providing an opportunity to increase their knowledge of inclusive midwifery care and LGBTQ+ health issues.

On the first page of the online questionnaires, all participants were informed in detail about the survey’s objective. A ‘click-if-you-agree’ button obtained informed consent. Participants were asked to provide their email address to enable matching of pre- and post-intervention questionnaire responses. While this information allowed for longitudinal data pairing, all survey responses were treated as strictly confidential. Identifiable information was stored separately from response data, and access was limited to authorized members of the research team. All data handling procedures complied with relevant data protection and ethical guidelines.

### Intervention and data collection

Midwives received a 3-hour, small-group evidence-based educational intervention (comprising a didactic lecture, case discussion, and group discussion) on gender equality, reproductive health rights, and reproductive and perinatal midwifery care for LGBTQ+ individuals. The structure, teaching methods, and learning objectives of the intervention are presented in Supplementary file Table S1. Pre- and post-surveys were administered to establish the baseline knowledge and attitudes, as well as the effectiveness of the intervention.

### Assessment of the intervention

The intervention was assessed at three time points: baseline (pre-intervention), immediately post-intervention, and follow-up at 6 months after the intervention to evaluate both short- and long-term effects.

The surveys consisted of three domains: 1) demographics, 2) an *ad hoc* basic knowledge assessment (twelve questions) about the reproductive health of LGBTQ+ people (knowledge score part 2), and 3) the Greek version^14^ of the original questionnaire developed by Rowe et al.^20^, as the Perception and Knowledge of Sexual and Gender Minority Health (PKSGMH) survey. More specifically, the survey included 81 questions regarding: 1) demographics (six questions on age, gender, sexual orientation and marital status); 2) former focused training in LGBT patient care (two questions); 3) attitudes toward LGBT non-patients (12 questions, 5-point Likert scale, with lower scores indicating more positive attitudes); 4) midwives’ attitudes toward LGBT patients (six questions, 5-point Likert scale, with lower scores indicating more positive attitudes); 5) Sexual and Gender Minority Affirmative Practice Scale (15 items with two subscales, 5-point Likert scale, with higher scores indicating greater cultural competence); 6) knowledge on LGBT Patients (13 true/false questions and nine true/false concerning lesbians, gays, bisexuals, transgender people summed to form total knowledge score); 7) inclusive patient care policy in health facilities (nine true/false questions and two open-ended questions); and 8) professional midwifery experience (seven questions on education level, years in profession, and work environment).

### Statistical analysis

Participants’ scores were tested for normality using the Kolmogorov-Smirnov criterion. Quantitative variables were expressed as mean and standard deviation (SD) or as median and interquartile range (IQR), while categorical variables were expressed as absolute and relative frequencies. More specifically, for each scale mentioned above, mean or total scores were computed at all three time points. Friedman’s test was used for score comparisons between baseline, immediately post-intervention and follow-up at 6 months measurements. Bonferroni correction was used to control for Type I error. Three participants did not complete the follow-up at 6 months assessment. Missing values were minimal (4.3%) and were handled using multiple imputation to retain the full sample (n=70) for longitudinal analyses^21^. All reported p-values are two-tailed. Statistical significance was set at p<0.05, and analyses were conducted using SPSS statistical software (version 27.0).

## RESULTS

Data from 70 midwives were collected and analyzed. A total of 67 participants (95.7%) completed the follow-up at 6 months assessment. Participants’ mean age was 42.1 years (SD=12.2), and 97.1% were women, as presented in Table 1. Most participants were heterosexual (87.1%), held a Bachelor’s degree (50.0%), and were married (62.9%). Also, 75.7% had LGBTQ+ friends, family members or acquaintances, and 18.6% had been trained to provide care to LGBTQ+ people.

**Table 1:** Sample characteristics (N=70)

*Characteristics*	*Categories*	*n*	*%*
**Gender**	Men	2	2.9
	Women	68	97.1
**Sexual orientation**	Heterosexual	61	87.1
	Homosexual	3	4.3
	Bisexual	6	8.6
**Degree**	Bachelor’s	35	50.0
	MSc	29	41.4
	PhD	6	8.6
**Marital status**	Married	44	62.9
	In a relationship	14	20.0
	Single	10	14.3
	Separated	2	2.9
**LGBTQ+ friends, family members or acquaintances**	No	17	24.3
	Yes	53	75.7
**LGBTQ+ people to whom care is provided**	Lesbians	41	58.6
	Gay men	7	10.0
	Bisexuals	16	22.9
	Transgender	6	8.6
**Had training in providing care to LGBTQ+ people**	No	57	81.4
	Yes	13	18.6
		***Mean***	***SD***
**Age** (years)		42.1	12.2
**Years from graduating**		18.0	12.7
**Years working as midwife**		16.0	12.5

### Attitudes towards LGBTQ+ people

**Figure 1:**
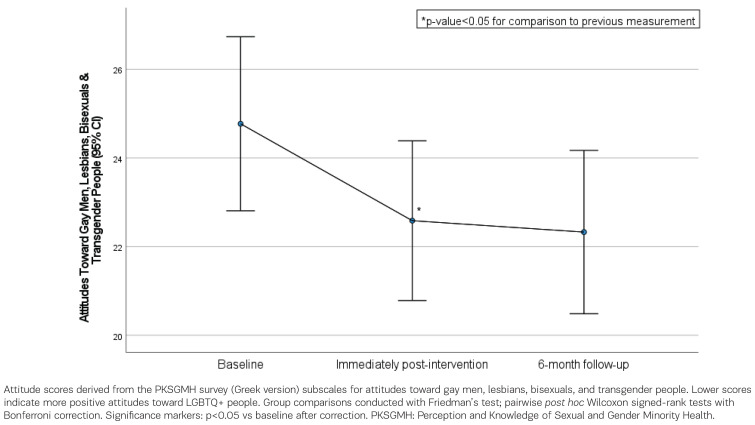
Participants’ attitude scores towards gay men, lesbians, bisexuals and transgender people, across the measurement time points (baseline, post-intervention, and follow-up at 6 months) (n=70 at baseline and post-intervention; n=67 at follow-up)

Participants’ attitude scores towards gay men, lesbians, bisexuals and transgender people are presented in Table 2, showing results for baseline (pre-intervention), immediately post-intervention, and follow-up at 6 months assessments. In all scores, significant changes occurred throughout the follow-up period. More specifically, after the Bonferroni correction, it was found that participants’ attitude scores towards gay men and lesbians significantly decreased from baseline to post-intervention (baseline 6.19 vs post-intervention 5.54, p=0.016, and baseline 6.14 vs post-intervention 5.59, p=0.011, respectively), indicating that they became more positive towards them. At the follow up at 6 months, this improvement was retained compared with the baseline (toward gay men: baseline 5.54 vs post-intervention 5.56, p>0.999; toward lesbians: baseline 5.59 vs post-intervention 5.74, p>0.999). After the Bonferroni correction, no significant difference was found among the three measurements. Participants’ attitudes towards transgender individuals also improved from baseline to post-intervention (baseline 6.3 vs post-intervention 5.71, p=0.093). Although there was no significant difference between post-intervention and 6-month follow-up scores (post-intervention 5.54 vs 6-month follow-up 5.51, p>0.999), their scores in comparison to the baseline measurement were significantly lower (baseline 6.3 vs follow-up at 6 months 5.51, p=0.034). Their total attitude score towards gay men, lesbians, bisexuals and transgender people decreased significantly from baseline to post-intervention (baseline 24.77 vs post-intervention 22.59, p=0.003) and remained at similar levels at follow-up at 6 months (post-intervention 22.59 vs follow-up at 6 months 22.33, p>0.999) (Figure 1).

**Table 2:** Participants’ attitude scores towards gay men, lesbians, bisexuals and transgender people, and changes across the measurement time points

	*Gay men*		*Lesbians*		*Bisexuals*		*Transgender people*		*All*	
	*Mean (SD)*	*Median (IQR)*	*Mean (SD)*	*Median (IQR)*	*Mean (SD)*	*Median (IQR)*	*Mean (SD)*	*Median (IQR)*	*Mean (SD)*	*Median (IQR)*
**Scores**										
Baseline	6.19 (2.12)	7 (4–8)	6.14 (2.27)	6 (4–8)	6.14 (2.27)	6.5 (4–8)	6.3 (2.11)	7 (5–8)	24.77 (8.23)	26.5 (17–31)
Post-intervention	5.54 (2.07)	6 (3–7)	5.59 (1.94)	6 (4–7)	5.74 (2.05)	6 (4–8)	5.71 (1.93)	6 (4–7)	22.59 (7.56)	24 (16–28)
Follow-up at 6 months	5.56 (2.22)	5 (4–7)	5.74 (2.16)	6 (4–7)	5.69 (2.05)	6 (4–7)	5.51 (1.91)	6 (4–7)	22.33 (7.73)	22.5 (16–28)
p (Friedman test)	**<0.001**		**0.001**		**0.027**		**0.006**		**<0.001**	
**Change**										
From baseline to post-intervention	-0.64 (1.84)	0 (-2–0)	-0.56 (1.78)	-0.5 (-1–0)	-0.4 (1.56)	0 (-1–0)	-0.59 (1.77)	0 (-2–0)	-2.19 (5.77)	-2 (-6–0)
p*	**0.016**		**0.011**		0.208		0.093		**0.003**	
From post-intervention to follow-up at 6 months	0.01 (1.77)	0 (-1–1)	0.16 (1.71)	0 (-1–1)	-0.06 (1.53)	0 (-1–1)	-0.2 (1.68)	0 (-1–1)	-0.26 (5.82)	0 (-3–4)
p*	>0.999		>0.999		>0.999		>0.999		>0.999	
From baseline to follow-up at 6 months	-0.63 (1.63)	-1 (-2–0)	-0.4 (1.4)	0 (-1–0)	-0.46 (1.62)	0 (-2–1)	-0.79 (1.84)	-0.5 (-2–0)	-2.44 (5.8)	-2.5 (-6–1)
p*	**0.016**		0.093		0.141		**0.034**		**0.016**	

Greater scores in attitude scores indicate more negative attitudes. IQR: interquartile range. *p-value for pairwise time comparisons after Bonferroni comparisons.

### Attitudes towards LGBTQ+ patients

**Figure 2:**
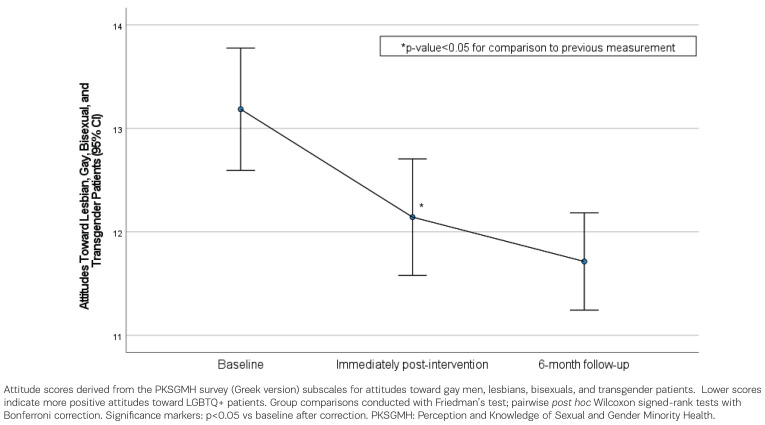
Participants’ attitude scores towards gay men, lesbians, bisexuals and transgender patients, across the measurement time points (baseline, post-intervention, and follow-up at 6 months) (n=70 at baseline and post-intervention; n=67 at follow-up)

Participants’ attitude scores towards gay men, lesbians, bisexuals and transgender patients are presented in Table 3, including baseline, post-intervention, and follow-up at 6 months measurements. Participants’ attitude score towards gay men, lesbians, bisexuals and transgender patients decreased significantly from baseline to post-intervention (baseline 13.19 vs post-intervention 12.14, p<0.001), indicating more positive attitudes, and remained significantly improved at the follow-up at 6 months (post-intervention 12.14 vs follow-up at 6 months 11.71, p>0.999) (Figure 2). At 6 months, their score continued to be significantly lower than baseline measurement.

**Table 3:** Participants’ attitude scores towards gay men, lesbians, bisexuals and transgender patients, scores in sexual and gender minority affirmative practice scale, knowledge scores, and changes across the measurement time points

	*Attitudes toward lesbian gay bisexual and transgender patients^a^*		*Sexual and gender minority affirmative practice scale^b^*		*Knowledge score^c^*		*Knowledge score part 2^c^*	
	*Mean (SD)*	*Median (IQR)*	*Mean (SD)*	*Median (IQR)*	*Mean (SD)*	*Median (IQR)*	*Mean (SD)*	*Median (IQR)*
**Scores**								
Baseline	13.19 (2.48)	13 (12–15)	67.01 (7.5)	69 (60–74)	10.61 (1.64)	11 (10–12)	5.26 (2.59)	6 (4–7)
Post-intervention	12.14 (2.36)	12 (11–14)	69.07 (6.83)	73 (60–75)	11.13 (1.27)	11.5 (11–12)	7.83 (1.47)	8 (7–9)
Follow-up at 6 months	11.71 (1.97)	11.5 (10–13)	68.56 (8.04)	72 (61–75)	11.31 (1.38)	12 (11–12)	7.51 (1.85)	8 (6–9)
p (Friedman test)	**<0.001**		**0.012**		**<0.001**		**<0.001**	
**Change**								
From baseline to post-intervention	-1.04 (2.36)	-2 (-3–0)	2.06 (6.37)	0.5 (0–3)	0.51 (1.35)	0 (0–1)	2.57 (2.74)	2 (1–4)
p*	**0.001**		**0.043**		**0.048**		**<0.001**	
From post-intervention to follow-up at 6 months	-0.43 (2.12)	0 (-2–1)	-0.51 (6.22)	0 (-2–1)	0.19 (1.3)	0 (0–1)	-0.31 (1.89)	0 (-2–1)
p*	>0.999		>0.999		>0.999		0.816	
From baseline to follow-up at 6 months	-1.47 (2.48)	-2 (-3–0)	1.54 (7.45)	0.5 (-1–4)				
p*	**<0.001**		0.189		**0.011**		**<0.001**	

a Greater scores in attitude scores indicate more negative attitudes.

b Greater scores in the sexual and gender minority affirmative practice scale indicate more positive practice when it comes to LGBTI+ patients.

c Greater knowledge scores indicate greater knowledge. IQR: interquartile range.

* p-value for pairwise time comparisons after Bonferroni comparisons.

### Affirmative practice scale

**Figure 3:**
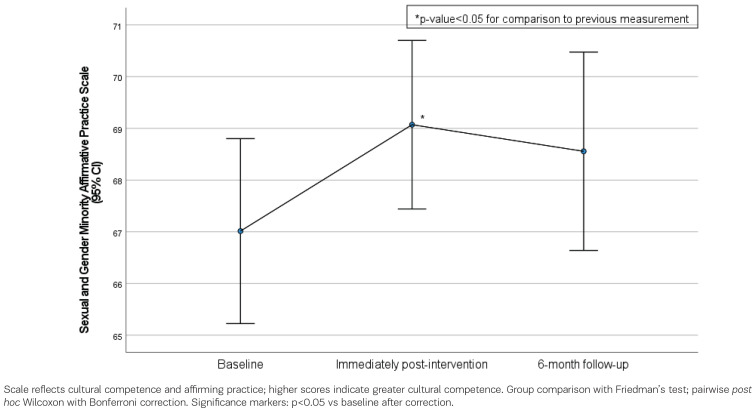
Participants’ scores in the Sexual and Gender Minority/Affirmative Practice Scale across the measurement time points (baseline, post-intervention, and follow-up at 6 months), (n=70 at baseline and post-intervention; n=67 at follow-up)

The participants’ scores on the Sexual and Gender Minority Affirmative Practice Scale are presented in Table 3, for baseline, post-intervention, and follow-up at 6 months assessments. This scale reflects the level of cultural competence, capturing midwives’ affirming attitudes and readiness to offer inclusive care, with higher scores indicating greater cultural competence. Participants’ scores on the Sexual and Gender Minority Affirmative Practice Scale increased significantly from baseline to post-intervention (baseline 67.01 vs post-intervention 69.07, p=0.043). At follow-up at 6 months, scores were maintained at similar levels to post-intervention (post-intervention 69.07 vs follow-up at 6 months 68.56, p>0.999), and remained higher than baseline, although the difference did not reach statistical significance (baseline 67.01 vs follow-up at 6 months 68.56, p=0.189) (Figure 3).

### Knowledge score

Both knowledge scores were significantly greater at post-intervention, in comparison to baseline measurements (baseline 10.61 vs post-intervention 11.13, p=0.048 for knowledge score; baseline 5.26 vs post-intervention 7.83, p<0.001 for knowledge score part 2) (Table 3). At the followup at 6 months, these scores continued to be significantly greater than those in the baseline (baseline 10.61 vs follow-up at 6 months 11.31, p=0.011 for knowledge score; baseline 5.26 vs follow-up at 6 months 7.51, p<0.001 for knowledge score part 2) and were at similar levels to those at the post-intervention measurement (post-intervention 11.13 vs follow-up at 6 months 11.31, p>0.05 for knowledge score; post-intervention 7.83 vs follow-up at 6 months 7.51, p>0.05 for knowledge score part 2).

## DISCUSSION

This longitudinal study of Greek midwives (n=70) demonstrated significant improvements in knowledge and attitudes toward LGBTQ+ individuals and patients after an educational intervention, with positive effects lasting for six months. More specifically, attitudes toward gay men and lesbians improved after the intervention and remained improved at the follow-up at 6 months. Attitudes toward transgender individuals also improved compared with baseline, while overall attitude scores toward LGBTQ+ individuals and patients decreased (more positive) post-intervention and were sustained at six months. Knowledge scores increased in both indices after training and remained higher at six months. Finally, the Sexual and Gender Minority Affirmative Practice scores, indicating cultural competence, increased immediately and were retained at follow-up.

Our findings align with international evidence which shows that targeted LGBTQ+ cultural-competency educational interventions and training improve providers’ knowledge, attitudes, self-efficacy and clinical skills across midwifery, nursing and allied health professions^2,8,11-13,22-24^. However, recent systematic reviews consistently emphasize the scarcity of long-term follow-up^23,24^, making the sustained six-month improvements observed in the present study a novel contribution of longitudinal data in the midwifery context, adding to reports of maintained gains up to 15 months in other healthcare-provider groups^23,25^.

The results of the present study also align with calls to embed LGBTQ+ health equity across midwifery continuing professional education^2,7^, by providing empirical support that even a brief, structured intervention can shift knowledge and attitudes and maintain these gains over time. Notably, evidence from New Zealand, Canada and the UK highlights the need for whole-of-program multidimensional approaches grounded in cultural safety, intersectionality and structural competence to challenge cis-heteronormativity and sustain long-term change in attitudes and clinical practice^2,3,7^. Between what is described as ideal and desirable, and the absence of any educational intervention, considering the well-documented gap in relevant education^26^, our findings encourage the development of even brief training programs.

A systematic review focused on midwifery services highlights that LGBTQ+ individuals often experience suboptimal midwifery care, subject to heteronormative assumptions, which results in feelings of discrimination, marginalization, and a lack of understanding of their distinct needs, findings leading the authors to conclude, among others, to the urgency of midwives’ education reform^27^. More recent systematic reviews in the midwifery field^15,24^, confirm previous findings^1,3,5-7^ that educational preparedness, through comprehensive and tailored education and training, is directly associated with more positive attitudes and greater readiness to deliver inclusive midwifery care, a key finding of the present study.

In Greece, the first part of the ATHENA Study provided the first national data on midwives’ knowledge and attitudes, by validating and using the PKSGMH instrument^14^ and identified significant training needs, limited exposure to LGBTQ+ health content and formal training. The present study, as the second part of the ATHENA Study, extends this work by demonstrating that targeted education can produce durable improvements in Greek midwives’ knowledge and attitudes toward LGBTQ+ individuals and LGBTQ+ health issues. It also provides a scalable model for integration into undergraduate and postgraduate curricula and continuing professional development, by underscoring the feasibility and necessity of implementing brief, evidence-based education at scale. Such initiatives, especially when supported by midwifery professional associations and national policy, hold the potential to dismantle structural cis-heteronormativity and promote equitable, gender-affirming reproductive and perinatal care across Europe^4,5,16,17,27^.

### Strengths and limitations

The present study has several strengths. First, the fact that a validated data collection tool^14^ was used increased the reliability of the findings. At the same time, the inclusion of two post-intervention assessments increased the validity of the findings, despite the relatively small sample size. Even in such a limited cohort, improvements in midwives’ knowledge and attitudes were demonstrated, highlighting the potential impact of a brief, structured educational intervention to produce sustainable and long-term effects. A further strength is the very high participant retention, with only three participants (4.3%) lost to the follow-up at 6 months across all study phases, an enabling condition that further reinforced the robustness of the longitudinal data and can be attributed to the voluntary nature of participation that might underlie participants’ presumed interest in the topic. At the same time, this presumed characteristic that these participants may have initially had a more favorable attitude toward the LGBTQ+ individuals limits the generalizability of the findings to the population of Greek registered midwives.

Furthermore, concerning the limitations of the study, this study used a single-arm pre-post design without a contemporaneous control, limiting causal inference and allowing for potential history or maturation effects. More importantly, because self-administered questionnaires were used, the results may be influenced by reporting biases, such as social desirability, where participants might consciously or unconsciously select responses that present themselves in a favorable or culturally acceptable way, potentially inflating positive changes in knowledge or attitudes. One further limitation of the present study is that the recruitment process, which relied on voluntary participation across three cities and required on-site participation, may have introduced selection bias and limited representativeness, thereby limiting the generalizability of the findings. Multiple imputation reduced attrition bias but rests on assumptions that may not fully hold. Finally, Bonferroni correction, while reducing Type I error, may have reduced power for some comparisons, such as in the variable ‘attitudes towards transgender individuals’.

Despite the limitations of the study that constrain the generalizability of the findings, the educational intervention of the study, which was characterized by a brief, evidence-based design delivered in small groups, was aligned with relevant international recommendations for curriculum implementation and continuing professional development programs, as it aimed not only to provide information and to build competencies in inclusive midwifery care, but also to reduce hostility and resistance toward the acceptance of sexuality and gender diversity^3,6,7^. Such an approach underscores its potential to be adapted across various national contexts and healthcare settings, as well as across different professional groups. Caution is warranted when extrapolating to settings with different health-system structures or to midwives who are less motivated or less interested in engaging in, or have limited access to, continuing professional development.

Future multi-site randomized or stepped-wedge studies with objective behavioral and patient-reported endpoints, and more extended follow-up periods (≥12 months), are needed to strengthen causal inference and clarify both the durability of the educational effects and the sustainability of the educational intervention.

## CONCLUSIONS

The study demonstrates that a brief, evidence-based, structured educational intervention can significantly enhance Greek midwives’ knowledge and attitudes toward LGBTQ+ individuals and patients, with positive effects maintained for at least six months. These findings provide rare longitudinal evidence of the sustainability of LGBTQ+ cultural-competency midwifery education and training and complement previous Greek baseline data that documented training needs but not retention of outcomes.

By showing that even a single three-hour educational session can lead to durable gains, the ATHENA Study underscores the value of incorporating LGBTQ+-inclusive content into midwifery education. Broader implementation of such interventions, supported by national policy and midwifery associations, has the potential to reduce heteronormative practices and promote equitable, gender-affirming reproductive and perinatal care for sexual and gender minority populations.

## Supplementary Material



## Data Availability

The data supporting this research are available from the authors on reasonable request.
