# Supplementary material for: The ATHENA Study: Evaluating the impact of an educational intervention on Greek midwives’ knowledge and attitudes toward LGBTQ+ reproductive and perinatal care
Source: Eur J Midwifery. 2025 Oct 31;9:10.18332/ejm/211383. doi: 10.18332/ejm/211383 (PMC12579269; doi:10.18332/ejm/211383)
Supplement: Supplementary file 1 [file EJM-9-48-s1.pdf]

Supplementary file Table 1. Structure, components, teaching methods, and learning objectives of the ATHENA Study educational intervention

Structure/Module	Teaching Methods	Learning Objectives
**Module 1. Fundamental concepts and terminology**<br>Definitions of sex, gender identity, gender expression, sexual orientation, and the LGBTQIA+ spectrum.	Didactic lecture (≈20 min) with PowerPoint slides and group Q&A.	• Define key terms related to gender and sexual diversity.<br>• Differentiate between biological sex, gender identity, and sexual orientation.<br>• Recognize the diversity of identities beyond binary classifications.
**Module 2. Epidemiology and health inequalities**<br>Overview of international and Greek data on LGBTQ+ health disparities, minority stress, and discrimination in healthcare.	Interactive lecture (≈15 min) with audience polling and examples.	• Describe main health disparities affecting LGBTQ+ individuals.<br>• Understand how stigma and structural discrimination impact health outcomes.
**Module 3. Stigma and discrimination in healthcare**<br>Historical context of pathologization, barriers to disclosure, implicit bias, and provider–patient communication issues.	Case-based discussion using real-world examples (≈20 min).	• Identify common forms of stigma and microaggressions in reproductive and perinatal care.<br>• Reflect on personal bias and communication style.
**Module 4. Health needs of LGBTQ+ individuals**<br>Sexual, reproductive, and mental health concerns; gender-based violence; access to fertility, contraception, and screening.	Didactic lecture and group discussion (≈25 min).	• Recognize key health priorities and risks across LGBTQ+ populations.<br>• Integrate inclusive, patient-centered approaches in midwifery care.
**Module 5. Inclusive midwifery care practices**<br>Gender-affirming communication, confidentiality, inclusive documentation, and	Role-play and group exercises (≈40 min) guided by facilitators.	• Apply inclusive language and behaviors in clinical interactions.<br>• Demonstrate gender-affirming care principles during simulated consultations.

supportive care for same-sex couples and transgender individuals.		
**Module 6. Reflection and integration** Group reflection, discussion of lessons learned, and action planning for clinical application.	Plenary debrief and Q&A (≈20 min).	• Consolidate key takeaways from the training.<br>• Develop strategies to implement inclusive practices in daily clinical settings.

Note: The 3-hour workshop was delivered in Greek in three cities (Athens, Thessaloniki, and Ptolemaida) in November 2024. All sites followed the same standardized curriculum and materials to ensure consistency across sessions.

© 2025 Antonakou A. et al.
